# Attempted altruistic infanticide in a context of psychotic decompensation induced by stress linked to the COVID-19pandemic: a case report

**DOI:** 10.1080/20961790.2021.1923138

**Published:** 2021-07-30

**Authors:** Camille Jantzi, Alexandre Perrin

**Affiliations:** University Centre of Legal Medicine (CURML), University of Geneva, Geneva, Switzerland

**Keywords:** Forensic sciences, forensic psychiatry, COVID-19, infanticide, psychosis, criminal responsibility, forensic evaluation

## Abstract

The mediatization of the COVID-19 pandemic has created a lot of stress leading sometimes to mental health issues. We present a case of a thirty-year-old woman with no history of psychotic disease but some vulnerabilities and no criminal record, who attempted to kill her seven-year-old son during a brief delusional episode in the context of fear of the coronavirus. She was successfully treated by pharmacotherapy and psychotherapy. She was examined by forensic psychiatrists leading to the conclusion that her responsibility was highly diminished, and her reoffending risk was low. We add to the literature that the COVID-19 pandemic has been such a stressor for mentally vulnerable people that it could lead to severe psychiatric decompensation and even criminal acts.

## Introduction

The COVID-19 pandemic has led to numerous public health consequences [1]. In terms of mental health there are two types of consequences. The firsts are directly caused by the effects of the infection, the coronavirus creating mental health symptoms, particularly psychotic symptoms [2,3]. The other consequences are in relation with the confinements, the restricted freedom of movement, the limited social contacts, the saturation of hospitalization capacities and a massive and alarming mediatization [4]. These last elements are important stress factors. They contributed to the emergence of psychiatric disease with psychotic, anxious or thymic symptoms, in people with or without history of mental illness [4–6]. Sometimes these symptoms have led to criminal or suicidal acts [7] including an increase in domestic violence caused by people suffering from a severe mental illness [8].

We report the situation of a woman presenting with an acute psychotic episode linked to the COVID-19 pandemic stress factors. This patient committed an attempted altruistic homicide against her son. She feared that he would be alone, thinking that she would soon die from the coronavirus. We present the forensic psychiatric evaluation she was submitted to, especially concerning her responsibility and dangerousness.

## Case report

### Anamnesis

The patient is a thirty-year-old woman, born in Slovakia, living in Switzerland since 2011. She is married but separated and involved in a divorce procedure. She grew up with both her parents and her two little brothers. She reported no history of psychiatric illness in her family. She was well treated, loved and cared for, without any financial problems or violence. She still has a good relationship with her family. She has experienced several traumatic events in her childhood. When she was 4 years old she got lost during a move. At the age of 14 she was sexually assaulted by a friend of her grandmother, forcefully kissing her in his car. When she was 16, she fell in a frozen lake and felt like she was dying but has been rescued quickly.

Concerning education, she finished high school without difficulties or behavioural issues. She had her first romantic relationship when she was between 16 and 20 years old. She qualifies this relationship as unhealthy, with a macho man who used to insult and diminish her regularly. During the last 2 years of this relationship, she experienced depressive symptoms like aboulia, anhedonia and sadness. She said she had to stop her studies because she could not concentrate enough anymore. She ended the relation by leaving the country for Italy where she fitted quickly in, having friends and finding a job. She met an older man, nice and soft, they became a couple but she did not feel in love with him and decided again to leave the country in order to end the relationship. She decided to settle in Switzerland where she was fast to find a social network and work as a home help for disabled people. She met the future father of her son, but the relationship was conflictual since the beginning. The patient showed some jealousy, suspecting that her partner still had intimate relations with his ex-girlfriend. After a year she got pregnant and they married. The pregnancy went on without complication, but the delivery still remains a traumatic memory for her; she describes it with vivid emotional reactions. She says that she had to endure a C-section against her will and was so agitated that her arms had to be tied up. She expresses feelings of disappointment, failure and fear. However, this event was not reported that way in her medical record. Back home she presented with anxiety towards her son’s health. She was so preoccupied he could suffer from sudden infant death syndrome that she had to sleep with him. The couple started then to sleep apart and their sexuality went impaired. She describes a fusional relation with her son, she was indeed still sleeping with him until the event, even though he was 7 years old. After the child’s birth she began to isolate from her previous social contacts, only wanting to go out in her son’s company. The relationship with her husband went worst; he began to have insulting and denigrating words towards her, a behaviour that her son tended to reproduce and against which she did not know how to react. She says that she was accepting it because family was the most important thing for her and must come before her own wellbeing. Since the beginning of 2019, the couple asked child protection services for help because their son presented with developmental issues and enuresis. At the same time her husband asked for divorce because he was seeing someone else for some months. The patient did not want this separation and hoped for the couple to reunite. At the beginning of the COVID-19 pandemic in Europe, she asked her husband to come back home with her and her son in order to protect them but he refused.

### The criminal offense

The homicidal attempt took place in March 2020, the day after Switzerland announced the establishment of the emergency state. Five days before, the Swiss government announced the strengthening of the public health measures like the closing of schools and the controls at borders. The patient felt more and more worried about the pandemic and feared to have caught the disease. She thought she would die and leave her son alone. She was listening to the news all day and even at night, having trouble to sleep. She began to act weirdly, stopped eating and ingested vinegar to disinfect herself. She ­developed delusional ideas, thinking that it was the end of the world and that all her family and friends were dead. She contacted her husband wanting to ask him to come in a hurry, but he did not reply; she then thought that he was dead too. She decided to strangle her son with her hands and with a cable, but the boy begged for his life, making her realize the seriousness of the offense and she stopped. She decided to isolate herself with her son in the nuclear shelter of her building. She stayed there until she was found by the police after her husband called them. When she saw him, she believed it was a hologram because she was still sure he was dead. She was led to the local psychiatric hospital where psychiatrists observed paranoiac and hypochondriac delusions with visual and auditory hallucinations, disorganisation, delusions of being controlled and imposed thoughts. She felt great guilt and sadness about the criminal acts. She was treated by Quetiapine and her symptoms disappeared in a few days. After a few weeks she asked to stop the treatment because she felt too tired and the psychiatrist agreed to reduce progressively the Quetiapine. It was stopped without any reoccurring of the psychotic symptoms, but she presented with anxious and depressive symptoms treated by psychotherapy and antidepressants. She then began to defensively rebuild her memory of the criminal act. She said that she never wanted to kill her son but to stimu­late his survival instinct to protect him. Anxiety and guilt calmed down, the patient now even presents indifference towards the distance with her son and towards the COVID-19 pandemic.

## Discussion

### Psychopathology

The patient presented with an acute psychotic disor­der in a stressful context. Nevertheless, the COVID-19 pandemic is a stressful situation for many people but not everyone will suffer from a psychotic decompensation. According to the stress-vulnerability model of Zubin and Spring [9], people suffering from mental illnesses would present different kind of vulnerabilities, innate or acquired (genetic, environmental, traumatic, etc.) and with various intensities. According to this theory a highly vulnerable person could suffer from a psychotic episode in reaction to a moderate level of stress whereas a high level of stress would be needed for a less vulnerable person [10]. This patient displays a fragile and anxious personality with borderline traits such as an altered self-image, seductive behaviours, excessive and inauthentic emotional expressions. We can also observe dependant traits like an exaggerated need for support, fusional relationships, abandonment anxiety, submissive behaviours, and excessive concerns about her family’s health. She experienced several traumatic episodes in her childhood. She also has a history of depression. The patient was overly sensitive to the alarming mediatization in the beginning of the COVID-19 pandemic in Europe. This anxious and dependant field planted the seeds of this psychotic decompensation, in a context of real abandonment represented by her divorce and imagined abandonment represented by the threat of contamination by the virus. A differential diagnosis could be a severe depression with psychotic symptoms because she had a history of depression and presented with depressive symptoms after being treated by Quetiapine. But she did not report depressive symptoms before the criminal offense and the symptoms afterwards were linked to her guilt vis-à-vis the homicidal attempt and to the context of detention. Furthermore, there was no manic symptoms indicating an entry into a bipolar disorder.

### Responsibility

In Switzerland, under Article 19 of the Penal Code, the responsibility of an offender can be diminished or even abolished if one was not able to understand the illicit nature of ones’ actions or to determine oneself under this comprehension. Psychiatric ­evaluations are mandated by the judge to enlighten these questions. When someone is penally irresponsible there is no sentence, but a medical care measure can be ordered. A diminished responsibility leads to an equally diminished sentence, the same care measures can be ordered.

In this case, the patient presented with an acute psychotic disorder in a context of stress linked to the COVID-19 pandemic. She suffered from delusional ideas; her perception of reality was highly severed. She still had a partial conscience of the seriousness of her actions, permitting herself to stop when her son begged. She felt immediate guilt and went to hide from the police. These delusional ideas initiated the criminal act, but the patient was able to partially control her behaviour. In the forensic psychiatric evaluation, we considered the responsibility of the patient to be highly restricted. Every psychotic episode does not necessarily lead to a penal irresponsibility and must be analysed under the specificity of the criminal act committed and the severity of the mental impairment.

### Dangerousness

The violence risk was assessed with a standardized tool, the HCR-20V3 scale (Historical-Clinical-Risk- Management Version 3) [11]. It is a checklist of 20 known risk factors of violence. In this case, the patient has few usual risk factors of violence. We can observe a relational instability, traumatic experiences in the past, an altered mental state, a limi­ted introspection and a high sensibility to stressful events. The risk of violence for this patient is causally linked to her mental state and thus to a new psychotic decompensation in the future. Risk factors of reoffending are then linked to risk factors of psychotic relapse. The first of these factors is stress; the patient shows a high sensitivity to stress and stressful life events are predictable for her such as divorce, the custody procedure, a physically and psychologically difficult work, the judgment of her acquaintances and solitude which is particularly hard for her since she presents with borderline and dependant personality traits. Furthermore, the COVID-19 pandemic is still active in Switzerland with contamination rates much higher than what they were at the moment of the criminal offense. Even if the patient claims to be preserved from mediatization in custody, it will not be the case when she goes out. Apart from these stress factors, there are other risk factors of psychotic relapse like an aggressive episode during the delusional decompensation or the presence of hallucinations [12]. The major issues of the medical care measure for the psychiatrist will be to detect early symptoms indicating a relapse and to adapt the treatment quickly, in contact with the probation services. The patient is willing to cooperate with the services and asking for psychiatric help. She wants to understand the circumstances that led her to commit the criminal act. She also has social and ­familial support, constituting an external control factor concerning reoffending. At the time of the evaluation, she had not seen her son since the offense. No contact was considered before the judgment. The reoffending risk was evaluated as low for violent acts in this context.

In this case, standardized evaluation tools and data from the scientific literature are considered less relevant considering the specificity of the criminal act and context. The altruistic infanticide is rare, and the standard evaluation tools are not designed specifically for women presenting with an acute psychotic episode leading to such an act. The psychotic episodes linked to the COVID-19 pandemic are only beginning to be described, it is not yet possible to know if their relapse rate is similar to other acute psychotic episodes. These patients need to be followed for several years to evaluate their evolution.

## Authors’ contributions

Camille Jantzi and Alexandre Perrin contributed equally to the initial expertise. Alexandre Perrin did the ­bibliography research and Camille Jantzi wrote the article.

## Compliance with ethical standards

No approval needed from the committee because it was not a research project.
